# Dr. Charles M. VanDyke

**Published:** 1914-12

**Authors:** 


**Dr. Charles M. VanDyke,** Starling of Columbus, O., 1885, died at his home in Himrod, September 29, aged 57.

